# A 12-week Taijiquan practice improves balance control and functional fitness in fall-prone postmenopausal women

**DOI:** 10.3389/fpubh.2024.1415477

**Published:** 2024-06-26

**Authors:** Xiaorong Bai, Wensheng Xiao, Kim Geok Soh, Yang Zhang

**Affiliations:** ^1^School of Physical Education, Huzhou University, Huzhou, China; ^2^Department of Sports Studies, Faculty of Educational Studies, Universiti Putra Malaysia, Serdang, Malaysia; ^3^Independent Researcher, Windermere, FL, United States

**Keywords:** exercise, falling, balance, flexibility, muscle strength, Tai Ji

## Abstract

**Purpose:**

Falls are the leading cause of accidental death among older persons, with postmenopausal women facing a greater hazard of falling due to osteoporosis. This study aimed to examine the effects of Taijiquan practice on balance control and functional fitness in at-risk females.

**Methods:**

Chinese women who self-reported a tendency to fall and had a baseline one-leg stand test time (4.1 s in the Taijiquan group) below the national average for their age group (60–64 years: 10.9 s, 65–69 years: 9.9 s) were assigned to either a control group (*n* = 26, mean age = 63.9 years) or a Taijiquan group (*n* = 24, mean age = 63.9 years). The Taijiquan group participated in a 12-week supervised intervention, while the control group maintained their daily activities. The average duration of each exercise session was 52 min. Static balance and functional fitness were assessed at the beginning and end of the intervention.

**Results:**

After 12 weeks, the Taijiquan group significantly outperformed the control group in terms of balance, flexibility, and muscular fitness (all *p* < 0.05). Participants in the Taijiquan group improved their one-leg stand by 61.0% (+2.5 s, Hedge’s *g* = 0.85), arm curl by 8.3% (+1.7 repetitions, *g* = 0.53), handgrip strength by 8.3% (+1.9 kg, *g* = 0.65), and sit-and-reach by 163.2% (+6.2 cm, *g* = 1.17).

**Conclusion:**

The improvement in balance, coupled with other functional fitness benefits, suggests that Taijiquan could serve as a useful exercise for older women with an elevated risk of falling.

## 1 Introduction

As of 2022, there were 771 million people aged 65 and older, and the proportion of the global population aged 65 and older is projected to increase from 10% in 2022 to 16% in 2050 (1). Age-related decline in balance is associated with an increased risk of falling (2). According to the WHO, one-third of people aged 65 and older fall at least once per year, with 5% of these falls resulting in a fracture (3). In China, the prevalence of falls, single falls, and recurrent falls was 26.4, 19.4, and 4.75%, respectively, among people over 65 years (4). Given China’s aging demographics, it is critical to explore preventive measures for reducing falls in vulnerable populations.

While strategies ranging from psychological counseling to environmental adaptations may be considered, regular exercise continues to be the primary focus of fall-prevention measures (5). The WHO’s 2020 physical activity guideline recommends that older persons pay particular attention to “functional balance and strength training” (6) to prevent falls and functional declines. Among many effective physical activities, Taijiquan—a traditional Chinese exercise—receives less public attention, even in China. According to four national surveys on popular physical activities among Chinese individuals, Taijiquan does not rank among the top 10 activities (7). Yet, available scientific evidence suggests that it is a useful exercise for older persons in terms of its various health benefits (8).

Research into Taijiquan’s benefits on balance control has burgeoned in the past two decades in China and other regions. Evidence to date suggests that Taijiquan appears to be a useful conditioning exercise for improving balance control among older persons experiencing functional decline (9). For this age cohort, exercise prescription should take account into both interventional efficacy and exercise safety. For example, while unilateral exercises or strength training on unstable surfaces are more effective in improving balance than bilateral exercises or training on stable surfaces (10, 11), these exercises are neither viable nor safe for vast majority of aging individuals. Given this consideration, low-aerobic dancing and Taijiquan are valuable in reducing the risk of falling in older persons (12).

Although a growing body of literature supports the efficacy of Taijiquan practice for balance control, there are two shortcomings. First, previous studies selected healthy individuals or those with already impaired physical functions (e.g., participants with knee surgery) to assess the balance control and health benefits of Taijiquan (13, 14). To date, while a few studies have examined Taijiquan’s benefits in reducing recurring falls (15), no study has investigated its effects on a cohort identified as having a high risk of falling but who have not experienced significant falls in the past. In a group of individuals aged 70 years or older who had fallen at least once in the preceding 12 months and had a healthcare practitioner’s referral indicating they were at risk of recurring falls, it was found that the risk of falling was reduced by 31% following 6 months of Taijiquan practice (15). Although this initial finding is encouraging, more evidence is needed to confirm the benefits across different demographics, particularly among those who have no significant fall history. For instance, a two-year longitudinal study indicated that while Taijiquan can prevent a decline in functional balance, its effectiveness in reducing injurious falls remains uncertain (16). In short, there is insufficient evidence to draw a definitive conclusion regarding Taijiquan’s benefits for older persons who are prone to falling. This may be attributed in part to the absence of a common definition of the risk of falling. Nevertheless, given that balance is a predictor of falls (17), those with poorer balance control than their age-matched peers may be categorized as a marginally fall-prone population. Hence, evaluating the effectiveness of Taijiquan practice among older persons at greater risk of falls could have important implications for fall prevention. Second, women, particularly postmenopausal women, tend to engage in less daily physical activity, which exacerbates age-related declines in balance, and the sharpest decline in balance has been seen in women between the ages of 60 and 70 (18). While there may not be a significant difference in the incidence of falls between Chinese males and females (19), it is important to note that postmenopausal women may suffer more severe consequences from accidental falls: postmenopausal women are more susceptible to osteoporosis, and, if they fall, they are more likely to sustain bone fractures (20). To address these concerns, previous research has shown that alternative forms of balance exercises, such as rocker-board and tandem stance activities, may be recommended for reducing falls (21). However, it is important to note that these exercises often require supervised instruction. For widespread promotion, home-based exercises are highly sought after (22). Nonetheless, it remains to be confirmed whether Taijiquan is an effective method for postmenopausal women. For instance, in a group of postmenopausal women with osteoporosis, 6 months of Taijiquan practice showed no significant effect on static balance control (23). Moreover, to date, no previous study has investigated the effects of Taijiquan practice on balance control among healthy postmenopausal women who are prone to falls.

Therefore, this study aimed to establish an evidence-based intervention using Taijiquan practice to improve the balance control of healthy, postmenopausal women prone to falls. We were also interested in if Taijiquan practice could improve functional fitness related to healthy aging. The overall objective was to offer empirical evidence regarding the effectiveness of Taijiquan in enhancing WHO’s call for “functional balance and strength” (6) among at-risk females.

## 2 Materials and methods

### 2.1 Participants

The research design was based on a cluster randomized controlled trial. The minimum sample size needed to detect a large effect size (Cohen’s *d* = 0.80) (24) with statistical significance (*α* = 0.05; 1-*β* = 0.8; two-tailed test) was calculated (G*Power) to be 52 individuals. Group assignments were not concealed from the study managers (site recruiters, Taijiquan coaches, and administrators of the evaluation) or participants. The researchers were blinded to group assignment until all statistical analyses were completed.

**Figure 1:**
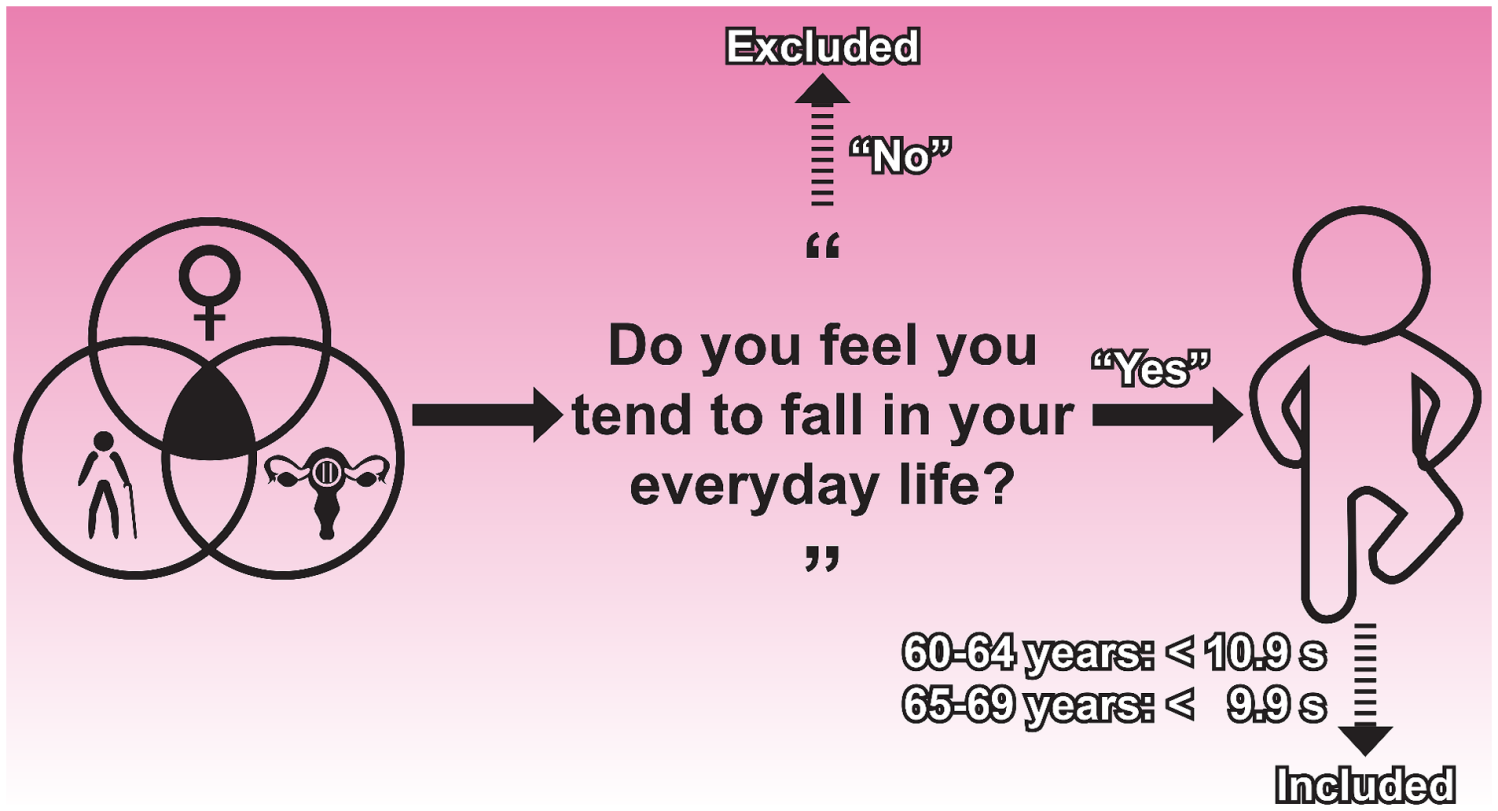
Recruiting fall-prone, postmenopausal women.

In the recruitment phase, defining a fall-prone individual was the most crucial step. Literature suggests that self-reported perceptions of fall risk can be exaggerated (25), raising doubts about their reliability. However, data from a prospective cohort study also indicated that assessments of fall risk should include both physiological and subjective measurements (26). In China, there are no national medical diagnostic criteria for fall risk. Due to this lack of consensus, we employed a two-step procedure to recruit a fall-prone cohort. Firstly, site recruiters distributed flyers in WeChat groups to potential participants residing in Puyang, Henan province. The flyers contained information about the study (e.g., non-invasive physical exercise training), participant eligibility criteria (detailed below), participant rights (e.g., free to withdraw from research at any time), obligations (e.g., adhering to all research requirements), potential risks (e.g., training-related injuries), and rewards (e.g., supervised 12-week training). A “yes-no” questionnaire asked, “Do you feel you tend to fall in your everyday life?” Only those who answered “yes” were contacted by the site manager. Next, potential participants underwent a one-leg stand test during an orientation session. Those with results below the age-specific national average (27) were invited to sign up for the 12-week study. The questionnaire was subjective yet simple to administer, while the one-leg stand test served as an objective predictor of falling in older persons (17). Both methods were well-suited to the purpose of this study. Figure 1 illustrates the two-step recruitment process.

The research was approved by the Ethics Committee of Universiti Putra Malaysia (protocol number: JKEUPM 2020–296). All participants provided written informed consent. The study included healthy women aged 60 to 69 years without known heart disease, hypertension, knee osteoarthritis, chronic obstructive pulmonary disease, musculoskeletal symptoms, or self-reported depression. Volunteers were excluded if they had a one-leg stand test result of less than 10.9 s for the 60–64 year-old age group or less than 9.9 s for the 65–69 year-old age group, had undergone surgery within the past year, participated in a structured exercise program within the past 6 months, used medications or nutritional supplements affecting blood pressure readings, were unwilling to consent to involuntary group assignment, or had experience with Taijiquan practice.

**Figure 2:**
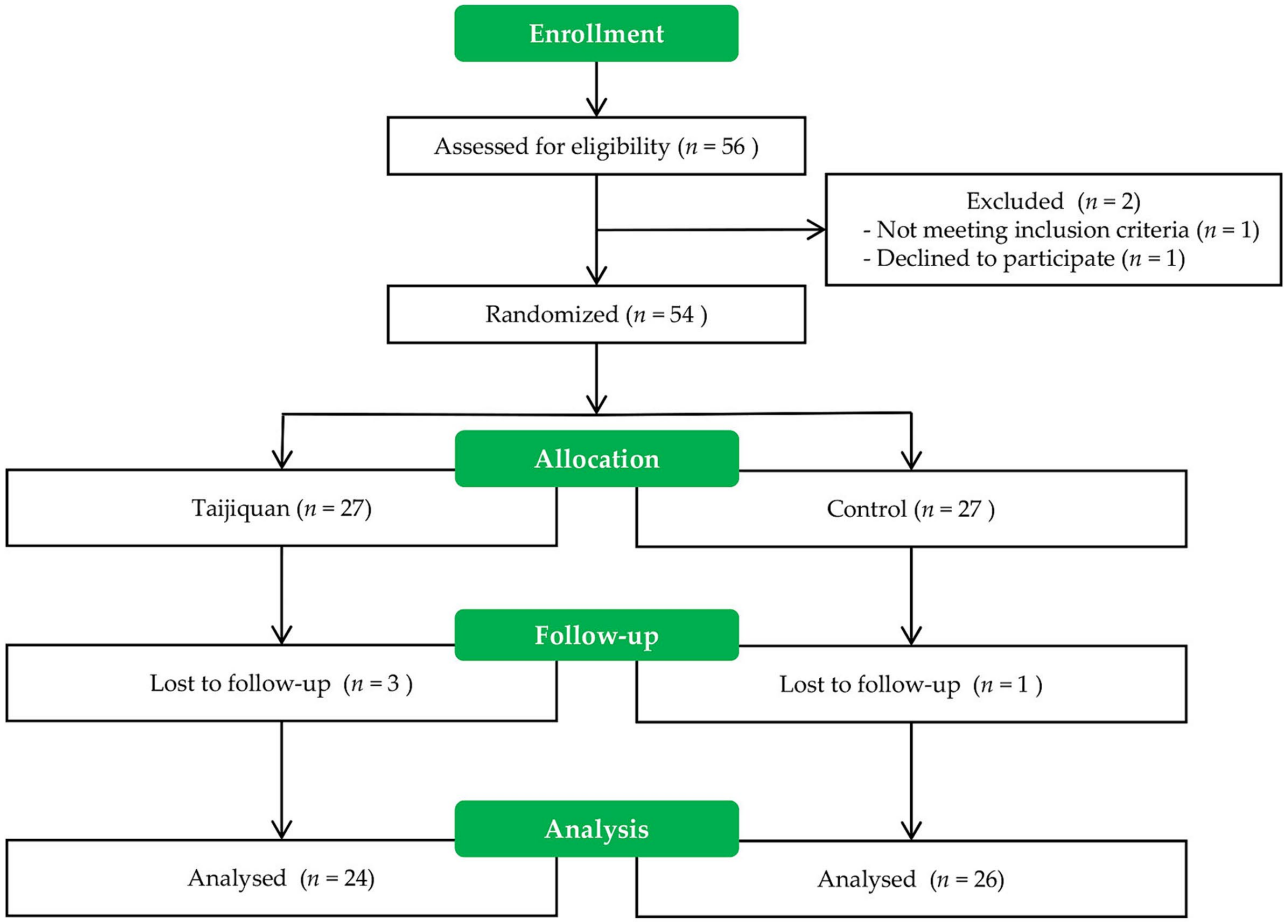
Study flow diagram.

Figure 2 illustrates the enrollment process. A total of 54 women met all criteria and were assigned to either the control or Taijiquan group. No exercise-related injuries occurred during the intervention. Three individuals in the Taijiquan group withdrew from the study (one due to travel and two due to relocation), while one individual in the control group withdrew due to a family situation. Consequently, data from 24 participants in the Taijiquan group and 26 participants in the control group were analyzed. Baseline demographic data are provided in Table 1.

**Table 1:** Demographic data.

Variable	Control	Taijiquan	*p*-value
Age, years	63.9 (2.9)	63.9 (3.1)	0.25
Height, cm	157.8 (4.6)	158.5 (5.0)	0.87
Weight, kg	65.8 (5.3)	64.9 (8.2)	0.43

Standard deviation in ().

### 2.2 Intervention

**Figure 3:**
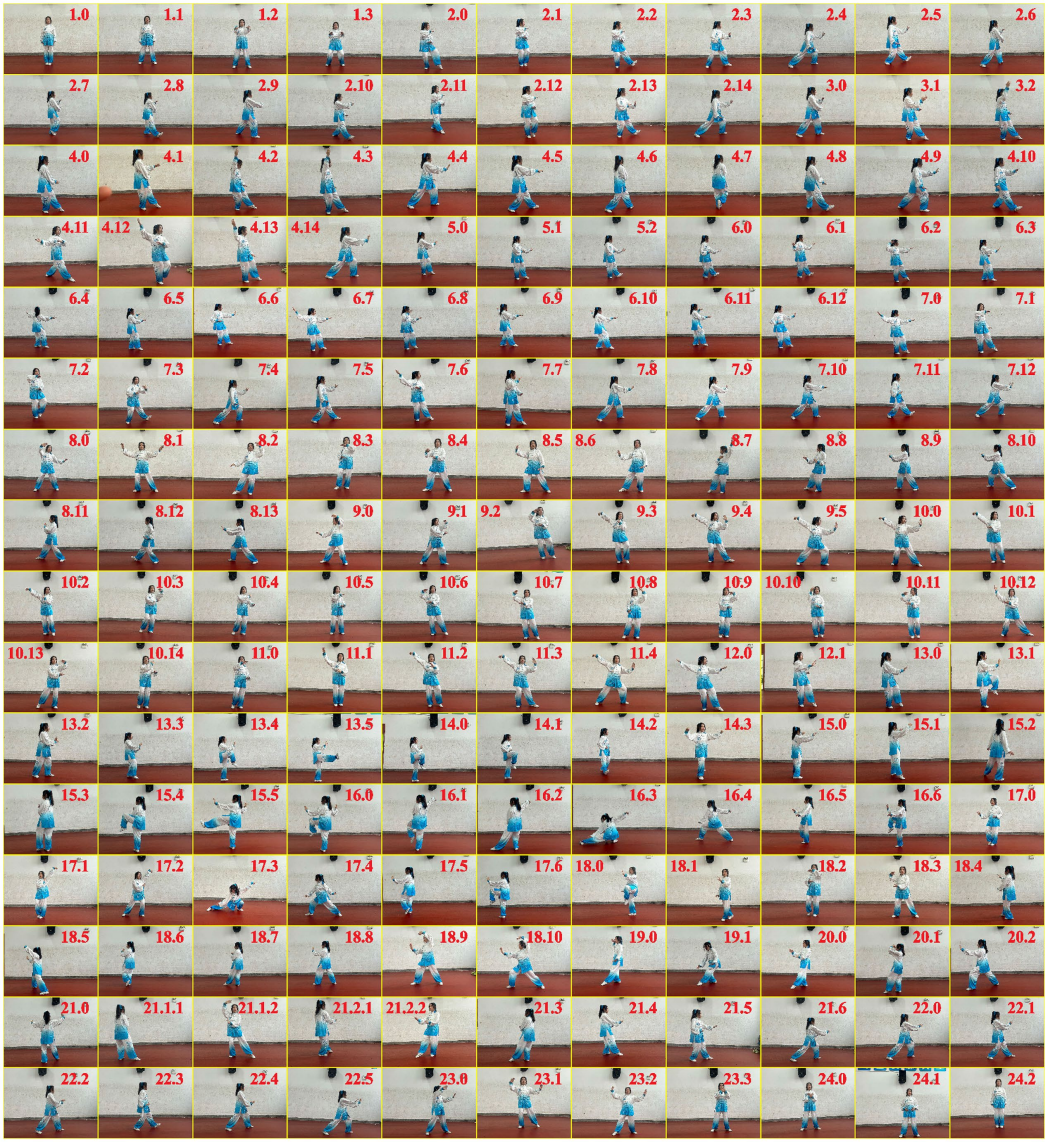
A Taijiquan master demonstrating the decomposed movements of the Yang-style 24-form Taijiquan. Readers are directed to the online version of the high-resolution photograph for detailed visualization (DOI: 10.6084/m9.figshare.22491958.v1).

The Taijiquan group underwent 12 weeks of supervised training at the Liutun Community in Puyang, taking place every Monday, Wednesday, and Friday from 6 to 7 a.m. The training sessions were supervised by one Taijiquan master and two trainees. Each session began with a 10-min warm-up, consisting of a 5-min slow marching activity followed by 5 min of stretching. The stretching routine included exercises such as neck flexion and extension, shoulder rotations, lateral arm swings, forward arm swings, wrist rotations, trunk rotations, forward leg swings, knee bends, and ankle rotations. Participants then practiced the Yang-style 24-form Taijiquan (refer to Figure 3) under the guidance of the Taijiquan master. As the participants were new to Taijiquan, the duration of Taijiquan sessions was gradually increased. In the first week, each session lasted 20 min. Every 2 weeks, the session duration was extended by 5 min until reaching 45 min by the 12th week. Each session concluded with a 10-min cool-down period. Participants engaged in a series of controlled deep breaths, repeating the process 10 times, followed by a comprehensive stretching routine targeting various muscle groups, including the quadriceps, calf, hamstring, upper body, triceps, shoulder, inner thigh, hip, and groin muscles. All physical activities were accompanied by calming music. Participants were advised against engaging in additional physical activities except for walking on non-training days. For the control group, no structured physical activity was allowed, and participants were free to engage in walking as part of their daily routine.

### 2.3 Evaluation

Participants underwent evaluations 2 days before the initial training session and 2 days after the final training session, conducted at 7 a.m. in the Liutun Community. Prior to the evaluations, participants were instructed to ensure they had sufficient sleep. All testing equipment was calibrated according to the manufacturer’s specifications, and the evaluation procedures for independent testers (six groups total, for each main test category) were standardized under the supervision of a qualified researcher (X.B.). The evaluations adhered to the National Physical Fitness Measurement Standards Manual (older adult Version) (28). Although the testing manual did not prescribe a specific sequence, health components requiring a resting state, such as blood pressure, were assessed first, followed by other functional fitness tests. In brief, the tests were administered as follows:

The one-leg stand with eyes closed was employed to evaluate balance control. Prior to the formal measurement, participants practiced the test position three to five times to familiarize themselves with it. They were instructed to start with a stable base of support and then stand unsupported, with eyes closed, on their dominant leg. The duration of the one-leg stand was timed from the moment the foot was lifted off the ground until it made contact again or the standing leg was compromised, using a stopwatch. A longer duration signifies better static balance.

Waist circumference, hip circumference, and waist-to-hip ratio were utilized to assess body composition. Measurements were taken using a measuring tape, with waist circumference measured midway between the lowest rib and the iliac crest. Hip circumference was measured at the widest point of the buttocks, ensuring the tape was parallel to the floor and not compressing the skin. Each participant’s waist and hip circumferences were measured three times, and the average of these measurements was used for data analysis.

To assess circulatory fitness, measurements were taken for blood pressure, resting heart rate, and forced vital capacity. An electronic sphygmomanometer (Omron HEM-7071A, Japan) was used to monitor diastolic blood pressure, systolic blood pressure, and resting heart rate after participants had been seated quietly for at least 5 minutes in a calm environment. Forced vital capacity was measured using spirometry equipment (Jianmin, GMCS-III type A, Xinheng Oriental Technology Development Co., Ltd., Beijing, China). Participants were instructed to exhale air from their lungs after taking a deep breath. This test was conducted three times, with the highest recorded value used for analysis.

Flexibility was assessed using the back scratch and sit-and-reach tests. In the back scratch test, participants stretched one hand over the shoulder and reached up to the center of the back with the other hand. The distance between their extended middle fingers was measured. For the sit-and-reach test, participants sat on the ground with bare feet touching and knees straight. Using their fingertips, they pushed a movable marker on a scale plate without bending their knees. The distance between the initial position and where the marker stopped was recorded. Measurements for both tests were recorded as positive or negative values.

Mental health was assessed using a Chinese-language Satisfaction with Life Scale (29). Participants rated the following statements on a 7-point Likert scale: “In most ways my life is close to my ideal,” “The conditions of my life are excellent,” “I am satisfied with my life,” “So far I have gotten the important things I want in life,” and “If I could live my life over, I would change almost nothing.” An overall score was derived by summing the responses to these five statements. Scores ranged from 5 to 35, with 20 representing the neutral point on the scale. Higher scores indicated greater life satisfaction.

Tests for muscular fitness comprised the arm curl, chair stand, and handgrip strength assessments. In the arm curl test, participants sat on a chair and performed arm bending movements with their dominant arm. The number of biceps curls completed in 30 s was recorded. In the chair stand test, participants executed full stands from a seated position with their arms folded across the chest, and the number of stands completed in 30 s was recorded. Handgrip strength was measured using a hand dynamometer, with participants instructed to exert maximal force using their dominant hand.

To ensure the reliability of the physical fitness measurements, we randomly selected 11 participants from each group to undergo a second physical assessment 1 day after the baseline evaluation. The retest was conducted at the same time of day as the initial assessment.

### 2.4 Statistics

The data were analyzed using IBM SPSS version 27, with a two-tailed alpha value of 0.05 considered statistically significant. Normality and equality of variances in the data were initially assessed using normalized quantile residuals and Levene’s test. For the primary analysis, a generalized estimating equation model was utilized, extending the logistic regression model for cluster randomized controlled trials (30). Interpretation of training effects was guided by gerontology-specific guidelines (31). Thresholds for small, medium, and large effects based on Hedges’ g were 0.16, 0.38, and 0.76, respectively. The reliability of the evaluation was assessed using the typical error measurement (32), and the smallest worthwhile changes corresponding to the three levels of effect size were also calculated.

## 3 Results

Given that the interpretation of the results could be influenced by the heterogeneity of the sample and the reliability of the measurement, we established the baseline measurement error and the smallest worthwhile change, as shown in Table 2. These study-level benchmark values are useful for determining the magnitude of the study-level effect sizes.

**Table 2:** Typical error measurement (TEM) and smallest worthwhile change (SWC) (*N* = 22).

Test battery	TEM	SWC_0.16_	SWC_0.38_	SWC_0.76_
One-leg stand with eyes closed (sec)	0.9	0.7	1.7	3.3
Hip circumference (cm)	0.5	1.0	2.5	4.7
Waist circumference (cm)	0.4	1.2	2.8	5.7
Diastolic blood pressure (mm Hg)	0.8	1.1	2.6	5.1
Systolic blood pressure (mm Hg)	2.1	0.8	1.8	3.6
Resting heart rate (bpm)	0.9	1.9	4.5	9.0
Forced vital capacity (mL)	179	95	226	451
Back scratch (cm)	1.2	1.3	3.1	6.3
Sit-and-reach test (cm)	1.0	1.5	3.6	7.1
Arm curl (rep)	1.2	0.8	1.8	3.6
Chair stand (num)	1.2	0.7	1.7	3.4
Handgrip strength (kg)	1.4	0.8	1.8	3.7

The subscript indicates the correponding effect size level.

As shown in Table 3, there were no notable changes in measurements for the control group after 12 weeks. Conversely, Taijiquan practice exhibited a large effect on the one-leg stand (+61.0%) and sit-and-reach test (+163.2%), a moderate effect on life satisfaction (+7.8%), arm curl (+8.3%), chair stand (+7.2%), and handgrip strength (+8.3%), and a small effect on diastolic blood pressure (−1.8%) and the back scratch (+48.3%). Using stricter criteria that take measurement errors into account, the effect size for the one-leg stand was medium, while the effect sizes for the arm curl and chair stand were small. Nevertheless, the Taijiquan group significantly outperformed the control group in the one-leg stand (*g* = 0.94), sit-and-reach test (*g* = 1.00), arm curl (*g* = 0.79), and handgrip strength (*g* = 0.86). These findings indicate that the 12-week Taijiquan practice significantly improved balance, flexibility, and upper-body muscular fitness among postmenopausal healthy women.

**Table 3:** Effects of the 12-week Taijiquan program on physical and mental health.

Test battery	Time	Measurement		Between-group		Within-group *g*	
		Control	Taijiquan	*p*-value	g	C	T
Balance (sec)	T0	3.7 (2.1)	4.1 (2.3)	1.00	0.17	0.04	0.85
	T12	3.8 (2.3)	6.6 (3.5)*	0.00	0.94		
	∆	0.1 (0.07) [2.7%]	2.5 (1.8) [61.0%]	-	-	-	-
**Body composition**							
HC (cm)	T0	102.2 (4.3)	101.8 (6.3)	1.00	0.06	0.01	0.03
	T12	102.2 (4.3)	101.6 (6.6)	1.00	0.10		
	∆	0 [0]	0.2 (0.1) [0.2%]	-	-	-	-
WC (cm)	T0	93.0 (6.8)	91.8 (6.7)	1.00	0.18	0.01	0.01
	T12	93.1 (6.9)	91.8 (7.1)	1.00	0.19		
	∆	0.1 (0.1) [0.1%]	0 [0]	-	-	-	-
WHR	T0	0.91 (0.04)	0.90 (0.03)	1.00	0.25	0.01	0.03
	T12	0.91 (0.04)	0.90 (0.03)	1.00	0.29		
	∆	0 [0]	0 [0]	-	-	-	-
**Circulatory health**							
DBP (mm Hg)	T0	74.4 (8.0)	76.4 (8.5)	1.00	0.24	0.01	0.16
	T12	74.4 (7.3)	75.0 (8.1)*	1.00	0.09		
	∆	0 [0]	−1.4 (1.0) [1.8%]	-	-	-	-
SBP (mm Hg)	T0	125.0 (6.8)	126.2 (5.8)	1.00	0.19	0.10	0.15
	T12	125.7 (6.3)	125.4 (6.1)	1.00	0.23		
	∆	0.7 (0.5) [0.6%]	−0.8 (0.6) [0.6%]	-	-	-	-
RHR (bpm)	T0	73.7 (8.4)	73.2 (8.4)	1.00	0.06	0.08	0.14
	T12	74.4 (7.9)	72.0 (8.3)*	1.00	0.29		
	∆	0.7 (0.5) [0.9%]	−1.2 (0.8) [1.6%]	-	-	-	-
FVC (mL)	T0	2,316 (475)	2,355 (364)	1.00	0.09	0.03	0.26
	T12	2,302 (419)	2,448 (348)	1.00	0.38		
	∆	−14 (9.9) [0.6%]	93 (65.8) [3.9%]	-	-	-	-
**Flexibility**							
BC (cm)	T0	−6.9 (9.5)	−6.0 (9.2)	1.00	0.10	0.01	0.33
	T12	−6.8 (9.1)	−3.1 (8.0)*	0.68	0.43		
	∆	0.1 (0.1) [1.4%]	2.9 (2.1) [48.3%]	-	-	-	-
SR (cm)	T0	3.8 (6.5)	3.8 (5.5)	1.00	0.01	0.03	1.17
	T12	4.0 (6.8)	10.0 (5.1)*	0.00	1.00		
	∆	0.2 (0.1) [5.3%]	6.2 (4.4) [163.2%]	-	-	-	-
Life satisfaction	T0	27.7 (3.6)	28.2 (3.4)	1.00	0.17	0.23	0.67
	T12	28.6 (4.4)	30.4 (3.1)*	0.44	0.49		
	∆	0.9 (0.6) [3.2%]	2.2 (1.6) [7.8%]	-	-	-	-
**Muscular fitness**							
AC (rep)	T0	19.7 (3.6)	20.4 (3.3)	1.00	0.21	0.02	0.53
	T12	19.6 (3.1)	22.1 (3.4)*	0.03	0.79		
	∆	−0.1 (0.1) [0.5%]	1.7 (1.2) [8.3%]	-	-	-	-
CS (num)	T0	17.6 (3.5)	18.1 (3.3)	1.00	0.16	0.13	0.39
	T12	18.0 (3.1)	19.4 (3.4)*	0.63	0.45		
	∆	0.4 (0.3) [2.3%]	1.3 (0.9) [7.2%]	-	-	-	-
HGS (kg)	T0	22.2 (3.6)	23.0 (3.1)	1.00	0.25	0.08	0.65
	T12	22.4 (3.0)	24.9 (2.8)*	0.01	0.86		
	∆	0.2 (0.1) [0.9%]	1.9 (1.3) [8.3%]	-	-	-	-

Standard deviation in () and percentage of delta—T12 - T0—in []. AC, arm curl; BS, back scratch; CS, chair stand; DBP, diastolic blood pressure; FVC, forced vital capacity; HC, hip circumferences; HGS, handgrip strength; RHR, resting heart rate; SBP, systolic blood pressure; SR, sit-and-reach test; T0, baseline evaluation; T12, 12-week evaluation; WC, waist circumferences; WHR, waist-to-hip ratio. *Within-group T0 vs. T12, *p* < 0.05.

## 4 Discussion

The recruitment of a group of healthy postmenopausal women prone to falling represents a novel aspect of this research. As aforementioned, there is no consensus on assessing low, medium, or high risk of falling. While the diagnostic precision of the two-step procedures employed in this study may be debatable, the importance of providing training in balance control to individuals with a fear of falling and below-average balance is undeniable. Considering this, the methodology employed to identify the at-risk population in this study may have broader applicability to other research focused on fall prevention. Following a 12-week intervention, the Taijiquan group demonstrated a 61.0% improvement in balance, an 8.3% enhancement in upper-body strength, and a remarkable 163.2% increase in flexibility. These gains not only helped participants achieve the expected goal of balance control but also improved handgrip strength beyond the national norm, potentially leading to long-term benefits for independent living and healthy aging.

Two noteworthy facts should be emphasized regarding the 61.0% improvement in balance control. First, the one-leg stand test serves as a reliable indicator of an individual’s susceptibility to falls (17), thus supporting the evidence-based eligibility criteria for our study’s primary objective. Second, a previous study showed that extending the duration of a one-leg stand by 1 s was associated with a 5% reduction in the risk of hip fractures (33). Our Taijiquan group achieved a noteworthy improvement of 2.5 s in this aspect, which can lead to substantial healthcare advantages. Hence, our study validates previous findings based on a broader population (34) and also contributes new knowledge regarding a target population (i.e., women who self-identified as prone to falling) and a target age range (i.e., 60 to 70 years) for early intervention (18). Additionally, compared to general balance training effects reported in the literature (Cohen’s *d* = 0.51) (35), our results suggest that Taijiquan is more effective. This reinforces our objective that Taijiquan is particularly valuable for older adults given its efficacy and saftey profiles. The mechanism underlying this improvement may be attributed to Taijiquan’s emphasis on rhythmic trunk rotation, forward-backward weight shifting, and gradual narrowing of the lower extremity stance (refer to Figure 3), which promotes neuromuscular adaptation for coordination and balance (36). Additionally, Taijiquan emphasizes the concept of “rooting with feet,” which involves ensuring heels make initial contact with the ground during all movements, whether circular or explosive (37). This practice is designed to enhance the fluidity of the center of body’s motion. As a result, improved normal gait and postural control can lead to enhancements in balance control.

Promoting appropriate balance exercises is a crucial consideration for long-term exercise participation and adherence (38), especially for older women at a higher risk of osteoporosis. Studies have shown that up to 40% of older persons who experience falls also have a pronounced fear of falling, leading to self-imposed restrictions on daily activities (39). This creates a negative feedback loop (falling-fear-inactivity) that can accelerate the aging process and increase the risk of premature morbidity and mortality. Taijiquan practice, with its few safety concerns and low-intensity physical activity, addresses these issues. Our findings highlight Taijiquan as a valuable exercise for older persons.

Despite the recruited participants having weak balance, their upper-body strength aligned with the national norm (27). Therefore, the gain in upper-body strength, particularly the improvement in handgrip strength [24.9 kg compared to the national norm of 23.6 kg for 60–64 years or 22.8 kg for 65–69 years (27)], was noteworthy and slightly exceeded what previous studies have shown (40). Western resistance theory typically emphasizes weights, repetitions, and volumes (41), whereas Taijiquan stands out as an outlier in this context: it is perceived as a gentle workout that does not require heavy loads, contact, or repetitive motions. The mechanism by which Taijiquan contributes to strength development is rooted in cultural heritage. Originally a martial art known as shadow boxing, Taijiquan was never intended as a relaxing activity but rather as an energy-intensive one. A study on Chen-style 56-form Taijiquan revealed a mean energy expenditure of 61.5 Kcal (42), corresponding to 10 Kcal/min of energy demand in the bare hands state during competitive routines lasting around 6 min. In comparison, a single set in a typical ACSM-style resistance training routine requires an average of 4.52 Kcal/min (43). Research by Hua and colleagues mapped the striking force of Chen-style Taijiquan (44), illustrating its impact on upper-body strength development. The philosophy of Taijiquan revolves around motion within stillness and overcoming hardness with softness. The improvement in upper-body muscular fitness is particularly relevant to aging, as arm curls are essential for daily activities, and low handgrip strength predicts all-cause mortality and cardiovascular disease (45). Therefore, Taijiquan, being an equipment-free form of strength training, is desirable for preserving functional fitness in home environments.

The ability of older persons to live independently is directly affected by age-related reductions in flexibility (46). In the Taijiquan group, participants increased their sit-and-reach distance from 3.8 cm to 10.0 cm, marking a significant improvement of 163.2%. Despite a baseline effect, Taijiquan improved this measurement from poor to well above the national norm (7.9 cm for 60–64 years and 7.1 cm for 65–69 years) (27), showcasing another remarkable outcome of this study. A previous cross-sectional study revealed that Taijiquan practitioners exhibited faster reflex reaction times in their hamstrings and gastrocnemius muscles, along with reduced knee joint angle repositioning errors (47). The forward and backward movements of the center of gravity during Taijiquan practice primarily involve increasing and decreasing the joint angles of the bilateral lower limbs, thereby strengthening the lower limb muscles (48). Contemporary Taijiquan, characterized by alternating slow motion and bursts of power, stimulates lumbar muscles (48) and enhances lower back flexibility and muscular strength (49). Consequently, improvements in hamstrings and lower back from Taijiquan practice are often accompanied by increased trunk flexibility. However, the absence of improvement in the back scratch test contradicts previous research findings (50). Given that the group means showed a significant difference in the single-arm portion of this study, it is likely that sample variation or study duration complicated the interpretation of results.

There were several insignificant outcomes that warrant attention. Firstly, the lack of development in lower-body strength may be attributed to baseline chair stand measurements being comparable to the national norm (27), which could have influenced subsequent lower-body strength gains in this medium-term study. However, a meta-analysis on the effect of exercise on NAD+ regulation suggests that multi-component training is more effective for healthy aging than a single form of exercise (51). In line with the WHO’s recommendation (6), we advocate for older persons to engage in various forms of physical activity alongside Taijiquan. For instance, incorporating walking into a Taijiquan session can target whole-body muscle groups and enhance overall physical fitness, especially beneficial for older persons with concurrent chronic conditions (52). Secondly, traditional Chinese cuisine is often rich in carbohydrates such as rice and noodles. With a steady rise in GDP *per capita*, modern Chinese households tend to consume excess calories, contributing to overweight (53). Therefore, sustained improvements in body composition require lifestyle modifications that include increased physical activity and consumption of nutrient-dense foods. Thirdly, although our findings contradict a previous study that reported Yang-style 24-form Taijiquan improved life satisfaction in healthy older persons (mean age 71.6 years) (54), we recommend that future studies continue to assess mental health outcomes. Interventions like Taijiquan may show greater sensitivity in assessment when mobility improves for individuals with existing movement impairments.

The absence of 24-h activity monitoring may have obscured the overall results. While participants were not allowed to engage in any exercise outside of the prescribed schedule during the study, they were permitted to walk freely. Unlike Western populations, Chinese individuals, especially the older adult, prioritize walking as their primary daily activity. Therefore, the lack of verification of step counts is a major drawback in interpreting the data. In addition, we only evaluated static balance as a measure of balance control. As we pointed out, the one-leg stand test is a proven reliable indicator of an individual’s susceptibility to falls (17). However, it would be valuable for future researchers to extend our findings by using other types of balance indicators, such as dynamic balance, reactive balance, and gait speed.

In conclusion, this study shows Taijiquan practice as a suitable exercise for postmenopausal women aiming to reduce their risk of falls. Considering the safety profile of Taijiquan, this conditioning exercise holds great relevance for aging societies in China. Moving forward, we contend that the research community has sufficiently demonstrated the modern health and fitness benefits of Taijiquan. However, despite its traditional roots, Taijiquan does not rank among the top 10 physical activities in China. We argue that the primary barrier to promoting this exercise lies in the lack of clear instructional guidance. Therefore, we recommend that Chinese policymakers prioritize the training of qualified social sports instructors and regularly introduce Taijiquan classes into communities. This approach will enable people to learn and experience the various benefits of Taijiquan. Meanwhile, Taijiquan has been endorsed by the United Nations as an intangible cultural heritage in 2020, highlighting its potential as a universal exercise practice that transcends cultural, linguistic, and geographical barriers. To overcome the main operational barrier of learning to practice this exercise, the Yang-style 24-form Taijiquan used in this study may be ideal for promotion in other nations. This form is simplified for easier practice and has demonstrated general efficacy in improving balance control compared to other forms of Taijiquan (55). Overall, our study contributes to the literature concerning a growing target population in China, and along with numerous other studies, it provides broader context of promoting Taijiquan as an aging-friendly exercise.

## Data availability statement

The raw data supporting the conclusions of this article will be made available by the authors, without undue reservation.

## Ethics statement

The studies involving humans were approved by this study was reviewed and approved by Ethics Committee of Universiti Putra Malaysia, with the approval number: JKEUPM 2020-296. The studies were conducted in accordance with the local legislation and institutional requirements. The participants provided their written informed consent to participate in this study. Written informed consent was obtained from the individual(s) for the publication of any potentially identifiable images or data included in this article.

## Author contributions

XB: Conceptualization, Data curation, Formal analysis, Methodology, Writing – original draft, Writing – review & editing. WX: Conceptualization, Data curation, Formal analysis, Writing – original draft. KS: Supervision, Writing – review & editing. YZ: Writing – review & editing.

## References

[1] United Nations. World population prospects 2022: Summary of results. New York: United Nations (2022).

[2] Rantakokko M Mänty M Rantanen T. Mobility decline in old age. Exerc Sport Sci Rev. (2013) 41:19–25. doi: 10.1097/JES.0b013e3182556f1e 23038241

[3] World Health Organization. Step safely: Strategies for preventing and managing falls across the life-course. Geneva: World Health Organization (2021).

[4] Chu L-W Chiu AYY Chi I. Falls and subsequent health service utilization in community-dwelling Chinese older adults. Arch Gerontol Geriatr. (2008) 46:125–35. doi: 10.1016/j.archger.2007.03.005 17467081

[5] Ganz DA Latham NK. Prevention of falls in community-dwelling older adults. N Engl J Med. (2020) 382:734–43. doi: 10.1056/NEJMcp1903252 32074420

[6] Bull FC Al-Ansari SS Biddle S Borodulin K Buman MP Cardon G. World Health Organization 2020 guidelines on physical activity and sedentary behaviour. Br J Sports Med. (2020) 54:1451–62. doi: 10.1136/bjsports-2020-102955, PMID: 33239350 PMC7719906

[7] Yang Y Jiang R Yuan F Zhao Y. A research on our mass sports development trends J Southwest China Normal. Univ Nat Sci Edu. (2017) 6:121–8. doi: 10.13718/j.cnki.xsxb.2017.06.021

[8] Wang C Collet JP Lau J. The effect of tai Chi on health outcomes in patients with chronic conditions: a systematic review. Arch Intern Med. (2004) 164:493–501. doi: 10.1001/archinte.164.5.493 15006825

[9] Chen W Li M Li H Lin Y Feng Z. Tai Chi for fall prevention and balance improvement in older adults: a systematic review and meta-analysis of randomized controlled trials. Front Public Health. (2023) 11:1236050. doi: 10.3389/fpubh.2023.1236050, PMID: 37736087 PMC10509476

[10] Behm DG Muehlbauer T Kibele A Granacher U. Effects of strength training using unstable surfaces on strength, power and balance performance across the lifespan: a systematic review and Meta-analysis. Sports Med. (2015) 45:1645–69. doi: 10.1007/s40279-015-0384-x, PMID: 26359066 PMC4656700

[11] Zhang W Chen X Xu K Xie H Li D. Effect of unilateral training and bilateral training on physical performance: A meta-analysis. Front Physiol. (2023) 14:1128250. doi: 10.3389/fphys.2023.1128250, PMID: 37123275 PMC10133687

[12] Mattle M Chocano-Bedoya PO Fischbacher M Meyer U Abderhalden LA Lang W. Association of Dance-Based Mind-Motor Activities with Falls and Physical Function among Healthy Older Adults: a systematic review and Meta-analysis. JAMA Netw Open. (2020) 3:e2017688–8. doi: 10.1001/jamanetworkopen.2020.17688, PMID: 32975570 PMC7519422

[13] Ghandali E Moghadam ST Hadian MR Olyaei G Jalaie S Sajjadi E. The effect of tai Chi exercises on postural stability and control in older patients with knee osteoarthritis. J Bodyw Mov Ther. (2017) 21:594–8. doi: 10.1016/j.jbmt.2016.09.001 28750970

[14] Aras B Seyyar GK Fidan O Colak E. The effect of tai Chi on functional mobility, balance and falls in Parkinson's disease: a systematic review and meta-analysis of systematic reviews. Explore. (2022) 18:402–10. doi: 10.1016/j.explore.2021.12.002 34952799

[15] Li F Harmer P Fitzgerald K Eckstrom E Akers L Chou L-S. Effectiveness of a therapeutic tai Ji Quan intervention vs a multimodal exercise intervention to prevent falls among older adults at high risk of falling: a randomized clinical trial. JAMA Intern Med. (2018) 178:1301–10. doi: 10.1001/jamainternmed.2018.3915, PMID: 30208396 PMC6233748

[16] Lin M-R Hwang H-F Wang Y-W Chang S-H Wolf SL. Community-based tai Chi and its effect on injurious falls, balance, gait, and fear of falling in older people. Phys Ther. (2006) 86:1189–201. doi: 10.2522/ptj.20040408, PMID: 16959668

[17] Vellas BJ Wayne SJ Romero L Baumgartner RN Rubenstein LZ Garry PJ. One-leg balance is an important predictor of injurious falls in older persons. J Am Geriatr Soc. (1997) 45:735–8. doi: 10.1111/j.1532-5415.1997.tb01479.x 9180669

[18] Daly RM Rosengren BE Alwis G Ahlborg HG Sernbo I Karlsson MK. Gender specific age-related changes in bone density, muscle strength and functional performance in the elderly: a-10 year prospective population-based study. BMC Geriatr. (2013) 13:71. doi: 10.1186/1471-2318-13-71, PMID: 23829776 PMC3716823

[19] Ye P Er Y Wang H Fang L Li B Ivers R. Burden of falls among people aged 60 years and older in mainland China, 1990–2019: findings from the global burden of disease study 2019. Lancet Public Health. (2021) 6:e907–18. doi: 10.1016/S2468-2667(21)00231-0 34838197 PMC8646839

[20] Peng K Tian M Andersen M Zhang J Liu Y Wang Q. Incidence, risk factors and economic burden of fall-related injuries in older Chinese people: a systematic review. Inj Prev. (2019) 25:4–12. doi: 10.1136/injuryprev-2018-042982 30670560

[21] Zouita S Zouhal H Ferchichi H Paillard T Dziri C Hackney AC. Effects of Combined Balance and Strength Training on Measures of Balance and Muscle Strength in Older Women With a History of Falls. Front Physiol. (2020) 11:619016. doi: 10.3389/fphys.2020.619016, PMID: 33424642 PMC7786296

[22] Costa SN Vieira ER Bento PCB. Effects of home- and center-based exercise programs on the strength, function, and gait of Prefrail older women: a randomized control trial. J Aging Phys Act. (2020) 28:122–30. doi: 10.1123/japa.2018-0363, PMID: 31629355

[23] Ferrara PE Salini S Maggi L Foti C Maccauro G Ronconi G. Evaluation of quality of life and static balance in postmenopausal osteoporosis women after tai Chi Chuan practice: an observational randomized case control study. J Biol Regul Homeost Agents. (2019) 33:163–9. 31172734

[24] Song R Ahn S So H Lee E-H Chung Y Park M. Effects of T'ai Chi on balance: a population-based Meta-analysis. J Altern Complement Med. (2015) 21:141–51. doi: 10.1089/acm.2014.0056, PMID: 25650522

[25] Dolan H Slebodnik M Taylor-Piliae R. Older adults' perceptions of their fall risk in the hospital: an integrative review. J Clin Nurs. (2022) 31:2418–36. doi: 10.1111/jocn.16125 34786777

[26] Delbaere K Close JCT Brodaty H Sachdev P Lord SR. Determinants of disparities between perceived and physiological risk of falling among elderly people: cohort study. BMJ. (2010) 341:c4165. doi: 10.1136/bmj.c4165 20724399 PMC2930273

[27] China Institute of Sport Science. (2021). Fifth national physical fitness monitoring bulletin china institute of sport science. Available at: https://www.sport.gov.cn/n315/n329/c24335066/content.html.

[28] General Administration of Sport of China. National Physical Fitness Measurement Standards Manual (elderly version). Beijing, China: People's Sports Publishing House (2003).

[29] Diener E Emmons RA Larsen RJ Griffin S. The satisfaction with life scale. J Pers Assess. (1985) 49:71–5. doi: 10.1207/s15327752jpa4901_13 16367493

[30] Peters T Richards S Bankhead C Ades A Sterne J. Comparison of methods for analysing cluster randomized trials: an example involving a factorial design. Int J Epidemiol. (2003) 32:840–6. doi: 10.1093/ije/dyg228 14559762

[31] Brydges CR. Effect size guidelines, sample size calculations, and statistical power in gerontology Innov. Aging. (2019) 3:1–8. doi: 10.1093/geroni/igz036, PMID: 31528719 PMC6736231

[32] Hopkins WG. Measures of reliability in sports medicine and science. Sports Med. (2000) 30:1–15. doi: 10.2165/00007256-200030010-00001 10907753

[33] Lundin H Sääf M Strender LE Nyren S Johansson SE Salminen H. One-leg standing time and hip-fracture prediction. Osteoporos Int. (2014) 25:1305–11. doi: 10.1007/s00198-013-2593-1 24562837

[34] Zhong D Xiao Q Xiao X Li Y Ye J Xia L. Tai Chi for improving balance and reducing falls: an overview of 14 systematic reviews. Ann Phys Rehabil Med. (2020) 63:505–17. doi: 10.1016/j.rehab.2019.12.008 31981834

[35] Lesinski M Hortobágyi T Muehlbauer T Gollhofer A Granacher U. Effects of balance training on balance performance in healthy older adults: a systematic review and Meta-analysis. Sports Med. (2015) 45:1721–38. doi: 10.1007/s40279-015-0375-y, PMID: 26325622 PMC4656699

[36] McGibbon CA Krebs DE Parker SW Scarborough DM Wayne PM Wolf SL. Tai Chi and vestibular rehabilitation improve vestibulopathic gait via different neuromuscular mechanisms: preliminary report. BMC Neurol. (2005) 5:3. doi: 10.1186/1471-2377-5-3, PMID: 15717934 PMC552306

[37] Jiménez-Martín PJ Meléndez-Ortega A Albers U Schofield D. A review of tai Chi Chuan and parameters related to balance. Eur J Integr Med. (2013) 5:469–75. doi: 10.1016/j.eujim.2013.08.001

[38] Williams DM. Exercise, affect, and adherence: an integrated model and a case for self-paced exercise. J Sport Exerc Psychol. (2008) 30:471–96. doi: 10.1123/jsep.30.5.471, PMID: 18971508 PMC4222174

[39] Ambrose AF Paul G Hausdorff JM. Risk factors for falls among older adults: a review of the literature. Maturitas. (2013) 75:51–61. doi: 10.1016/j.maturitas.2013.02.009 23523272

[40] Wang C Liang J Si Y Li Z Lu A. The effectiveness of traditional Chinese medicine-based exercise on physical performance, balance and muscle strength among older adults: a systematic review with meta-analysis. Aging Clin Exp Res. (2022) 34:725–40. doi: 10.1007/s40520-021-01964-2, PMID: 34420189

[41] Scott BR Duthie GM Thornton HR Dascombe BJ. Training monitoring for resistance exercise: theory and applications. Sports Med. (2016) 46:687–98. doi: 10.1007/s40279-015-0454-0 26780346

[42] Cai J Wei W Hua S Lin Z. Research and analysis on exercise intensity and energy consumption of single set of high and low rack Chen(56)tai-jiquan Fujian sports. Sci Technol. (2022) 41:55–8. doi: 10.3969/j.issn.1004-8790.2022.02.011

[43] Phillips WT Ziuraitis JR. Energy cost of the ACSM single-set resistance training protocol. J Strength Cond Res. (2003) 17:350–5. doi: 10.1519/00124278-200305000-00023, PMID: 12741877

[44] Hua H Zhu D Wang Y. Comparative study on the joint biomechanics of different skill level practitioners in Chen-style tai Chi punching. Int J Environ Res Public Health. (2022) 19:5915. doi: 10.3390/ijerph19105915, PMID: 35627452 PMC9141462

[45] Leong DP Teo KK Rangarajan S Lopez-Jaramillo P Avezum A Jr Orlandini A. Prognostic value of grip strength: findings from the prospective urban rural epidemiology (PURE) study. Lancet. (2015) 386:266–73. doi: 10.1016/S0140-6736(14)62000-6, PMID: 25982160

[46] Bravell ME Zarit SH Johansson B. Self-reported activities of daily living and performance-based functional ability: a study of congruence among the oldest old. Eur J Ageing. (2011) 8:199–209. doi: 10.1007/s10433-011-0192-6, PMID: 28798650 PMC5547338

[47] Fong S-M Ng GY. The effects on sensorimotor performance and balance with tai Chi training. Arch Phys Med Rehabil. (2006) 87:82–7. doi: 10.1016/j.apmr.2005.09.017 16401443

[48] Chan SP Luk TC Hong Y. Kinematic and electromyographic analysis of the push movement in tai chi. Br J Sports Med. (2003) 37:339–44. doi: 10.1136/bjsm.37.4.339, PMID: 12893721 PMC1724683

[49] Wang R Chang X-L Kiartivich S Wang X-Q. Effect of tai Chi Quan on the pressure pain thresholds of lower Back muscles in healthy women. J Pain Res. (2022) 15:403–12. doi: 10.2147/JPR.S353465, PMID: 35173478 PMC8842641

[50] Taylor-Piliae RE Newell KA Cherin R Lee MJ King AC Haskell WL. Effects of tai Chi and Western exercise on physical and cognitive functioning in healthy community-dwelling older adults. J Aging Phys Act. (2010) 18:261–79. doi: 10.1123/japa.18.3.261, PMID: 20651414 PMC4699673

[51] Sun X Su L Bu T Zhang Y. Exercise training upregulates intracellular nicotinamide phosphoribosyltransferase expression in humans: a systematic review with meta-analysis. Front Public Health. (2023) 11:1287421. doi: 10.3389/fpubh.2023.1287421, PMID: 37954044 PMC10639164

[52] Xiao W Wang B Bai X Tang S Zhang Y. Taoist way of a balanced exercise training cocktail for the management of primary hypertension in older persons. Front Public Health. (2023) 11:1308375. doi: 10.3389/fpubh.2023.1308375, PMID: 38155893 PMC10754045

[53] Pan X-F Wang L Pan A. Epidemiology and determinants of obesity in China. Lancet Diabetes Endocrinol. (2021) 9:373–92. doi: 10.1016/S2213-8587(21)00045-0 34022156

[54] Oh C Kang H. Effects of tai Chi exercise on the body composition, self-efficacy and life satisfaction of older adults in Korean local community. Int J Gerontol. (2019) 13:134–8. doi: 10.6890/IJGE.201906_13(2).0007

[55] Lin J Ning S Lyu S Gao H Shao X Tan Z. The effects of different types of tai Chi exercises on preventing falls in older adults: a systematic review and network meta-analysis. Aging Clin Exp Res. (2024) 36:65. doi: 10.1007/s40520-023-02674-7, PMID: 38472538 PMC10933200

